# Supplementary material for: Genome-wide association mapping of total antioxidant capacity, phenols, tannins, and flavonoids in a panel of Sorghum bicolor and S. bicolor × S. halepense populations using multi-locus models
Source: PLoS One. 2019 Dec 5;14(12):e0225979. doi: 10.1371/journal.pone.0225979 (PMC6894842; doi:10.1371/journal.pone.0225979)
Supplement: S1 Table — Highlighted in green are genes annotated from Rhodes et al. 2014,2017, in orange genes annotated as similar to Peroxidase, in yellow new annotations from sorghum genome in Atlas. In the first three columns start and stop position on the sorghum genome and transcript name, followed by the nearest marker name and the distance of the gene from the nearest marker, then a column where are shown the GWAS methods and target traits for which the linked SNP was significant, the last column shows the category of the genes. (DOCX) [file pone.0225979.s001.docx]

**Table S1. Annotated genes harboring major effect markers (R^2^ ≥ 15%). Highlighted in green are genes annotated from Rhodes et al. 2014,2017, in orange genes annotated as similar to Peroxidase, in yellow new annotations from sorghum genome in Atlas. In the first three columns start and stop position on the sorghum genome and transcript name, followed by the nearest marker name and the distance of the gene from the nearest marker, then a column where are shown the GWAS methods and target traits for which the linked SNP was significant, the last column shows the category of the genes.**

| Start | Stop | Gene name | Gene product | Nearest marker | Distance (Kb) | Method: trait | Gene category |
| --- | --- | --- | --- | --- | --- | --- | --- |
| 60,601,837 | 60,599,425 | Sobic.001G317800.1 | similar to glutathione S-transferase, GST | Chr1_61095994 | 494 | FarmCPU: FEN, TAC,  SUPER: TAN | Transport |
| 60,622,662 | 60,621,797 | Sobic.001G317900.1 | similar to Glutathione-S-transferase - BZ2 (GRMZM2G016241) | Chr1_61095994 | 473 | FarmCPU: FEN, TAC,  SUPER: TAN | Transport |
| 60,645,212 | 60,644,084 | Sobic.001G318000.1 | similar to Glutathione-S-transferase - BZ2 (GRMZM2G016241) | Chr1_61095994 | 451 | FarmCPU: FEN, TAC,  SUPER: TAN | Transport |
| 60,654,357 | 60,652,885 | Sobic.001G318200.1 | similar to Glutathione-S-transferase - BZ2 (GRMZM2G016241) | Chr1_61095994 | 442 | FarmCPU: FEN, TAC,  SUPER: TAN | Transport |
| 60,656,158 | 60,655,125 | Sobic.001G318300.1 | similar to Glutathione-S-transferase – BZ2 (GRMZM2G016241) | Chr1_61095994 | 440 | FarmCPU: FEN, TAC,  SUPER: TAN | Transport |
| 60,665,068 | 60,663,308 | Sobic.001G318366.1 | similar to glutathione S-transferase, GST | Chr1_61095994 | 431 | FarmCPU: FEN, TAC,  SUPER: TAN | Transport |
| 60,668,479 | 60,666,903 | Sobic.001G318432.1 | similar to glutathione S-transferase, GST | Chr1_61095994 | 428 | FarmCPU: FEN, TAC,  SUPER: TAN | Transport |
| 60,671,880 | 60,673,032 | Sobic.001G318500.1 | similar to Glutathione-S-transferase - BZ2 (GRMZM2G016241) | Chr1_61095994 | 424 | FarmCPU: FEN, TAC,  SUPER: TAN | Transport |
| 60,678,263 | 60,676,363 | Sobic.001G318600.2 | similar to glutathione S-transferase, GST | Chr1_61095994 | 418 | FarmCPU: FEN, TAC,  SUPER: TAN | Transport |
| 60,679,315 | 60,678,466 | Sobic.001G318700.1 | similar to Glutathione-S-transferase - BZ2 (GRMZM2G016241) | Chr1_61095994 | 417 | FarmCPU: FEN, TAC,  SUPER: TAN | Transport |
| 60,698,308 | 60,696,764 | Sobic.001G318800.1 | similar to Glutathione-S-transferase - BZ2 (GRMZM2G016241) | Chr1_61095994 | 398 | FarmCPU: FEN, TAC,  SUPER: TAN | Transport |
| 60,703,732 | 60,702,173 | Sobic.001G318900.1 | similar to Glutathione-S-transferase - BZ2 (GRMZM2G016241) | Chr1_61095994 | 392 | FarmCPU: FEN, TAC,  SUPER: TAN | Transport |
| 60,717,351 | 60,716,016 | Sobic.001G319000.1 | similar to Glutathione-S-transferase - BZ2 (GRMZM2G016241) | Chr1_61095994 | 379 | FarmCPU: FEN, TAC,  SUPER: TAN | Transport |
| 60,718,433 | 60,717,647 | Sobic.001G319100.1 | similar to Glutathione-S-transferase - BZ2 (GRMZM2G016241) | Chr1_61095994 | 378 | FarmCPU: FEN, TAC,  SUPER: TAN | Transport |
| 60,720,933 | 60,719,814 | Sobic.001G319200.1 | similar to Glutathione-S-transferase - BZ2 (GRMZM2G016241) | Chr1_61095994 | 375 | FarmCPU: FEN, TAC,  SUPER: TAN | Transport |
| 60,726,408 | 60,725,293 | Sobic.001G319300.2 | similar to glutathione S-transferase, GST | Chr1_61095994 | 370 | FarmCPU: FEN, TAC,  SUPER: TAN | Transport |
| 60,742,198 | 60,743,267 | Sobic.001G319500.1 | similar to Glutathione-S-transferase - BZ2 (GRMZM2G016241) | Chr1_61095994 | 354 | FarmCPU: FEN, TAC,  SUPER: TAN | Transport |
| 60,746,115 | 60,747,280 | Sobic.001G319600.1 | similar to Glutathione-S-transferase - BZ2 (GRMZM2G016241) | Chr1_61095994 | 350 | FarmCPU: FEN, TAC,  SUPER: TAN | Transport |
| 60,752,843 | 60,753,967 | Sobic.001G319700.1 | similar to Glutathione-S-transferase - BZ2 (GRMZM2G016241) | Chr1_61095994 | 343 | FarmCPU: FEN, TAC,  SUPER: TAN | Transport |
| 60,861,939 | 60,860,230 | Sobic.001G320900.1 | similar to MULTIDRUG RESISTANCE PROTEIN | Chr1_61095994 | 234 | FarmCPU: FEN, TAC,  SUPER: TAN | Transport |
| 14,236,989 | 14,235,462 | Sobic.002G115700.1 | similar to Putative chalcone synthase, CHS - TT4 (AT5G13930) | Chr2_13905455 | 332 | FarmCPU: TAN | Biosynthesis |
| 49,417,919 | 49,419,821 | Sobic.004G156100.2 | similar to Peroxidase / Lactoperoxidase | Chr4_48609207 | 809 | FarmCPU:TAC | Oxidation |
| 59,079,614 | 59,078,044 | Sobic.004G242600.1 | similar to peroxidase | Chr4_59458260 | 379 | FarmCPU:FEN  SUPER: FEN | Oxidation |
| 59,093,179 | 59,091,869 | Sobic.004G242900.1 | similar to WD40 repeat protein | Chr4_59458260 | 365 | FarmCPU:FEN  SUPER: FEN | Regulation |
| 59,576,464 | 59,578,405 | Sobic.004G248700.1 | similar to Myb-like DNA-binding protein | Chr4_59458260 | 118 | FarmCPU:FEN  SUPER: FEN | Regulation |
| 60,238,034 | 60,247,841 | Sobic.004G256500.1 | similar to WD40 repeat protein | Chr4_60134605 | 103 | FarmCPU:FEN, TAC  SUPER: FEN, FLA, TAC | Regulation |
| 60,330,551 | 60,336,650 | Sobic.004G257400.1 | similar to WD40 repeat protein | Chr4_60363744 | 33 | FarmCPU:FEN, TAC  SUPER: FEN, FLA, TAC | Regulation |
| 60,509,946 | 60,506,445 | Sobic.004G260000.1 | similar to Flavonol synthase | Chr4_60405075 | 105 | SUPER: FEN, FLA, TAC  FarmCPU:TAC | Biosynthesis |
| 61,143,019 | 61,137,552 | Sobic.004G267000.2 | similar to Myb-like DNA-binding protein | Chr4_61104509 | 39 | SUPER: FEN, FLA, TAC, TAN  FarmCPU: FLA, TAC | Regulation |
| 61,192,356 | 61,195,289 | Sobic.004G267800.1 | similar to Leucoanthocyanin reductase (LAR) - VvLAR1 (GSVIVG01011958001) | Chr4_61104509 | 88 | SUPER: FEN, FLA, TAC , TAN  FarmCPU: FLA, TAC | Biosynthesis |
| 61,268,235 | 61,270,278 | Sobic.004G268500.1 | similar to 9-cis-epoxycarotenoid dioxygenase / beta carotene dioxygenase | Chr4_61104509 | 164 | SUPER: FEN, FLA, TAC, TAN  FarmCPU: FLA, TAC | Oxidation |
| 61,483,970 | 61,480,770 | Sobic.004G270600.1 | similar to Myb-like DNA-binding protein | Chr4_61591217 | 107 | SUPER: FEN, FLA, TAC, TAN  FarmCPU: FLA | Regulation |
| 61,493,534 | 61,495,588 | Sobic.004G270900.3 | similar to Basic helix-loop-helix (BHLH) transcription factor | Chr4_61591217 | 98 | SUPER: FEN, FLA, TA , TAN  FarmCPU: FLA | Regulation |
| 61,497,409 | 61,488,123 | Sobic.004G270800.3 | similar to WD40 repeat protein - TTG1 (AT5G24520) | Chr4_61591217 | 94 | SUPER: FEN, FLA, TAC, TAN  FarmCPU: FLA | Regulation |
| 61,636,902 | 61,641,337 | Sobic.004G272700.1 | similar to 4-coumarate - CoA ligase, 4CL | Chr4_61636303 | 1 | SUPER: FEN, FLA, TAC, TAN  FarmCPU: FLA | Biosynthesis |
| 61,677,115 | 61,671,482 | Sobic.004G273000.1 | similar to Myb-like DNA-binding protein | Chr4_61636303 | 41 | SUPER: FEN, FLA, TAC, TAN  FarmCPU: FLA | Regulation |
| 61,765,784 | 61,767,930 | Sobic.004G273800.1 | similar to MYB transcription factor - ZM1, TT2 (GRMZM5G833253) | Chr4_61636303 | 129 | SUPER: FEN, FLA, TAC, TAN  FarmCPU: FLA | Regulation |
| 62,802,656 | 62,792,662 | Sobic.004G285400.5 | similar to Zn-finger transcription factor -TT1 (AT1G34790) | Chr4_63531227 | 729 | SUPER: FLA,TAC, FEN, TAN | Regulation |
| 64,224,364 | 64,225,877 | Sobic.004G303400.1 | similar to Myb-like DNA-binding protein | Chr4_64019027 | 205 | SUPER: FEN, FLA, TAN, TAC | Regulation |
| 64,256,534 | 64,253,228 | Sobic.004G303600.1 | similar to Myb-like DNA-binding protein | Chr4_64019027 | 238 | SUPER: FEN, FLA, TAN, TAC | Regulation |
| 5,610,250 | 5,612,213 | Sobic.007G055100.1 | similar to Myb-like DNA-binding protein | Chr7_5827884 | 218 | FarmCPU: FEN | Regulation |
| 5,637,300 | 5,639,394 | Sobic.007G055300.2 | similar to Myb-like DNA-binding protein | Chr7_5827884 | 191 | FarmCPU: FEN | Regulation |
| 5,813,802 | 5,813,065 | Sobic.007G056400.2 | similar to H+-ATPase proton pump | Chr7_5827884 | 14 | FarmCPU: FEN | Transport |
| 6,114,305 | 6,107,597 | Sobic.007G058900.1 | similar to Chalcone synthase | Chr7_5827884 | 286 | FarmCPU: FEN | Biosynthesis |
| 6,205,567 | 6,203,931 | Sobic.007G059200.2 | similar to Isoflavone-7-O-beta-glucoside 6''-O-malonyltransferase / Flavone/flavonol 7-O-beta-D-glucoside malonyltransferase | Chr7_5827884 | 378 | FarmCPU: FEN | Biosynthesis |
| 6,213,184 | 6,217,885 | Sobic.007G059400.1 | similar to Putative Anthocyanin 5-aromatic acyltransferase | Chr7_5827884 | 385 | FarmCPU: FEN | Biosynthesis |
| 57,642,054 | 57,642,731 | Sobic.007G146500.1 | similar to Myb-like DNA-binding protein | Chr7_58057317 | 415 | SUPER: FLA | Regulation |
| 57,974,505 | 57,976,114 | Sobic.007G148900.1 | similar to Flavone 3'-hydroxylase (F3'H) - TT7 (AT5G07990) | Chr7_58057317 | 83 | SUPER: FLA | Biosynthesis |
| 57,982,444 | 57,980,822 | Sobic.007G149000.1 | similar to Flavone 3'-hydroxylase (F3'H) - TT7 (AT5G07990) | Chr7_58057317 | 75 | SUPER: FLA | Biosynthesis |
| 61,910,539 | 61,914,508 | Sobic.007G186200.1 | similar to Putative anthocyanin-related membrane protein 1 | Chr7_62284152 | 374 | SUPER: FEN, FLA, TAN | Transport |
| 62,502,279 | 62,505,522 | Sobic.007G192300.1 | similar to peroxidase | Chr7_62396856 | 105 | SUPER: FEN, FLA, TAN | Oxidation |
| 62,595,684 | 62,587,839 | Sobic.007G193300.1 | similar to MADS-box transcription factor - TT16 (AT5G23260) | Chr7_62396856 | 199 | SUPER: FEN, FLA, TAN | Regulation |
| 62,650,085 | 62,660,122 | Sobic.007G193900.1 | WD40 REPEAT PROTEIN | Chr7_62396856 | 253 | SUPER: FEN, FLA, TAN | Regulation |
| 1,549,684 | 1,551,811 | Sobic.009G016600.1 | similar to Myb-like DNA-binding protein | Chr9_1550093 | 0 | SUPER: FLA | Regulation |
| 1,556,843 | 1,558,610 | Sobic.009G016633.1 | similar to Myb-like DNA-binding protein | Chr9_1550093 | 7 | SUPER: FLA, FEN | Regulation |
| 1,863,702 | 1,858,987 | Sobic.009G020900.1 | similar to H+-ATPase proton pump | Chr9_1550093 | 314 | SUPER: FLA | Transport |
| 2,039,570 | 2,040,733 | Sobic.009G023050.1 | similar to GLUTATHIONE S-TRANSFERASE, GST | Chr9_1550093 | 489 | SUPER: FLA | Transport |
| 48,493,990 | 48,477,458 | Sobic.009G131300.1 | similar to H+-ATPase proton pump | Chr9_48196807 | 297 | FarmCPU: FLA | Transport |
| 10,257,926 | 10,259,640 | Sobic.010G106601.1 | similar to Myb-like DNA-binding protein | Chr10_9810260 | 446 | FarmCPU: TAN | Regulation |
| 49,892,288 | 49,898,836 | Sobic.010G169000.1 | similar to MRP anthocyanin transporter ZmMRP3ZmMRP3 (GRMZM2G111903) | Chr10_50169631 | 271 | SUPER: FLA | Transport |
| 50,169,740 | 50,166,260 | Sobic.010G170300.1 | similar to Beta-glucosidase | Chr10_50169631 | 0 | SUPER: FLA | Oxidation |
