# Supplementary material for: Genome-wide association mapping of total antioxidant capacity, phenols, tannins, and flavonoids in a panel of Sorghum bicolor and S. bicolor × S. halepense populations using multi-locus models
Source: PLoS One. 2019 Dec 5;14(12):e0225979. doi: 10.1371/journal.pone.0225979 (PMC6894842; doi:10.1371/journal.pone.0225979)
Supplement: S2 Table — (DOCX) [file pone.0225979.s002.docx]

**Table S2. ‘A posteriori’ genes** **harboring major effect marker (R^2^ ≥ 15%)**

| Chr | Start | Stop | Transcript | Annotation | Marker in the gene |
| --- | --- | --- | --- | --- | --- |
| 2 | 13,901,450 | 13,929,533 | Sobic.002G113900.1 | similar to Putative CLB1 protein (Calcium-dependent lipid binding) protein | Chr2_13905455 |
| 4 | 60,362,942 | 60,368,204 | Sobic.004G257800.1 | similar to Putative chloride channel protein | Chr4_60363744 |
| 4 | 60,406,802 | 60,402,907 | Sobic.004G258400.1 | similar to Putative nucleoid DNA-binding protein cnd41 | Chr4_60405036; Chr4_60405075 |
| 4 | 61,615,234 | 61,617,221 | Sobic.004G272300.1 | similar to Putative uncharacterized protein | Chr4_61616880 |
| 4 | 63,531,196 | 63,532,671 | Sobic.004G295500.1 | similar to Putative AP2 domain containing protein | Chr4_63531227 |
| 4 | 63,899,808 | 63,901,322 | Sobic.004G300200.2 | uncharacterized | Chr4_63901177 |
| 4 | 64,018,712 | 64,019,678 | Sobic.004G301300.1 | similar to RING-H2 FINGER PROTEIN ATL45-RELATED | Chr4_64019027 |
| 7 | 5,827,165 | 5,830,578 | Sobic.007G056600.1 | similar to Putative uncharacterized protein | Chr7_5827884 |
| 7 | 58,057,524 | 58,047,095 | Sobic.007G149550.1 | Similar to REPLICATION PROTEIN A 70 KDA DNA-BINDING SUBUNIT C-RELATED | Chr7_58057317 |
| 7 | 62,284,724 | 62,279,160 | Sobic.007G190000.1 | similar to Putative uncharacterized protein | Chr7_62284152 |
| 9 | 48,199,194 | 48,195,000 | Sobic.009G128400.1 | similar to Putative uncharacterized protein | Chr9_48196807 |
